# Differences in biological behaviors between young and elderly patients with colorectal cancer

**DOI:** 10.1371/journal.pone.0218604

**Published:** 2019-06-18

**Authors:** Chinock Cheong, Seung Yeop Oh, Young Bae Kim, Kwang Wook Suh

**Affiliations:** 1 Department of Surgery, Ajou University School of Medicine, Suwon, Korea; 2 Department of Pathology, Ajou University School of Medicine, Suwon, Korea; University of Nebraska Medical Center, UNITED STATES

## Abstract

**Background:**

We investigated the differences in biological behaviors of sporadic colorectal cancer (CRC) between young and elderly patients. CRC is a common cancer, with a mean age at onset of > 65 years. However, recent reports indicate increasing rates in younger populations. The biological behaviors of sporadic CRC in elderly patients could differ from those in young patients.

**Methods:**

Between September 2007 and August 2012, we selected 723 CRC patients from our institution. The patients were divided into Group Y (n = 127, aged ≤50 years) and Group O (n = 596, aged >50 years). The clinicopathologic and oncologic outcomes in the two groups were compared.

**Results:**

Group Y tumors were characterized by higher incidences of mucin production (13.4% vs. 6.7%; *P* = 0.017), high microsatellite instability (MSI-H) (19.8% vs. 5.2%; *P* < 0.001), and N2 stage (32.3% vs. 22.1%; *P* = 0.020) than those in Group O. The recurrence rates were similar in both groups (14.9% vs. 17.3%; *P* = 0.665). The 5-year overall survival and disease-free survival did not differ. Multivariate analysis indicated that cellular differentiation and pathologic stage were significant prognostic factors for 5-year overall survival.

**Conclusion:**

Although age was not a prognostic factor for overall survival and young patients did not show a worse prognosis, there were differences in mucin production, MSI-H, and N2 stage between the two groups. Further studies are needed to clarify the clinical and biological characteristics of CRC, improve its treatment strategies, and promote better outcomes in young patients.

## Introduction

Colorectal cancer (CRC) is one of the most common cancers in Western Europe and the United States and its incidence has also been markedly increasing in developing countries such as Korea [1]. In general, patients aged > 65 years comprise the majority of the population with CRC [2]. However, recent reports indicate its increasing incidence in younger populations. Siegel et al. reported a decreasing incidence of CRC in individuals aged ≥ 50 years and an increase in those aged < 50 years between 2000 and 2013 [2]. In hereditary CRC, the disease onset is at a young age (approximately 45 years) [3]. However, the incidence of sporadic CRC in young patients is increasing [4,5].

We hypothesized that the biological behaviors of sporadic CRC occurring in elderly patients could differ from those in young patients. Since CRC is derived from multiple genetic mutations that lead to its development, sporadic CRC is generally considered a disease affecting the elderly. Therefore, this hypothesis has gained attention. Based on these previous observations, the prognosis of CRC in young patients may be worse than that in elderly patients. Because cancer cells in young patients seem to be more aggressive than those in elderly patients, young age itself had been postulated as a factor associated with worse prognosis [6–8]. Some authors have reported a higher incidence of advanced stage [6] and poorly differentiated carcinoma [7] as well as a shorter survival rate in young patients [7,9–12]. Recent studies involving large numbers of patients, however, observed no significant difference in survival rates between young and elderly groups [13–15]. These studies have shown conflicting data regarding the characteristics of young CRC patients, and the criteria used to differentiate between young and elderly patients are also ambiguous. Because there are few studies on young CRC patients in Korea, studies on this topic may be helpful for improving treatment strategies in this population.

The present study examined if the patient age affected the prognosis in cases of sporadic CRC. We compared the ratios of mucin production, cellular differentiation, and microsatellite instability (MSI) in addition to survival and clinicopathological features. The aim of this study was to identify the differences in clinical and biological features between young and elderly patients with sporadic CRC.

## Materials and methods

### Patients

Among patients with CRC treated in our institution between September 2007 and August 2012, 723 were included in this study. The selection criteria were as follows: patients who had undergone curative resection for primary CRC and who did not have any clinical evidence of hereditary CRC. Data on clinical and histopathologic features of CRC were collected from the database of our institution. This study was approved by the Institutional Review Board of Ajou Hospital (AJIRB-MED-MDB-18-330). We chose 50 years of age to divide patients into two groups and classified the patients as “elderly” or “young”. Patients aged ≤ 50 years were assigned to the “young group” (Group Y, n = 127), while those aged >50 years were assigned to the “elderly group” (Group O, n = 596). In the pathologic report, mucinous adenocarcinoma of colorectum was defined as an adenocarcinoma, of which >50% of the lesion is composed of extracellular mucin. We reported non-mucinous adenocarcinoma according to well, moderately or poorly differentiation and mucinous adenocarcinoma separately. We compared all clinicopathologic features including MSI status as well as treatment outcomes such as recurrence and survival rates in these two groups.

MSI analysis was conducted as follows. After DNA isolation from paraffin-embedded specimens, MSI analysis was performed using Bethesda microsatellite panel D2S123, D5S346, D17S250, BAT25, and BAT26. One primer from each primer pair had a 5’ fluorescent tag. Polymerase chain reaction (PCR) amplification was performed using a total volume of 25 μL containing 25–100 ng of DNA, 250 μmol/L of dexoynucleotide, 35–55 ng of primers, and 2 U of Amplitaq Gold (Applied Biosystems, Foster City, CA, USA). PCR was carried out at 94°C (30 seconds), 56°C (45 seconds), and 72°C (30 to 90 seconds) for 35 cycles (5 minutes initial denaturation and 7 minutes final elongation) using a GeneAmp PCR 2400 system (Applied Biosystems). The amplified fragments were visualized using capillary electrophoresis on an ABI 310 Genetic Analyzer (Applied Biosystems) and the microsatellite patterns of the tumors were compared to those of normal tissue specimens. According to the National Cancer Institute criteria, tumors with instability in two or more of the tested microsatellite loci were classified as MSI-H, while those with instability in one of the tested markers were considered low-frequency microsatellite instable low-frequency (MSI-L). Tumors with stability in all five of the tested loci were categorized as having microsatellite stability (MSS).

After curative resection, 5-fluorouracil-based adjuvant chemotherapy was administered to patients with stage III and high-risk stage II (perforated or obstructing cancer, perivascular invasion, T4 lesion, or poorly differentiated histology) disease.

### Statistical analysis

Two-tailed χ^2^ or Fisher’s exact tests were used to analyze categorical data. Continuous data were compared using Student t tests. Overall survival, disease-free and progression-free survival were calculated using the Kaplan–Meier method. Overall survival was calculated from the date of surgery to the date of death from CRC. Disease-free survival was calculated from the date of surgery to the date of recurrence or death from stage I, II, or III CRC. Progression-free survival was calculated from the date of surgery to the date of progression or death due to disease in stage IV CRC patients. Differences in survival were assessed by log-rank tests. The prognostic significance of clinicopathological factors was evaluated by multivariate analysis using Cox proportional hazards models. Statistical analysis was performed using IBM SPSS Statistics for Windows, version 20.0 (IBM Corp., Armonk, NY, USA). *P* values ≤0.05 were considered statistically significant.

## Results

### Pathological characteristics

The mean ages of the patients in Groups Y and O were 42.4 and 66.1 years, respectively. The tumors in Group Y were characterized by higher incidences of mucin production (13.4% vs. 6.7%; *P* = 0.017), MSI-H (19.8% vs. 5.2%; *P* < 0.001), and N2 stage (32.3% vs. 22.1%; *P* = 0.020) than those in Group O. The mean number of retrieved lymph nodes in the resected specimens from Group Y was higher than that in specimens from Group O (31.3 ± 26.1 vs. 21.6 ± 13.8; *P* < 0.001). The other clinicopathologic features in the two groups did not differ significantly (Table 1).

**Table 1:** Comparisons of the clinicopathological features between Groups Y and O with colorectal cancer.

Characteristics	Group Y (n = 127)	Group O (n = 596)	P value
Gender			0.842
Male	74 (58.3%)	355 (59.6%)	
Female	53 (41.7%)	241 (40.7%)	
Preoperative CEA			0.372
<5 ng/mL	82 (67.8%)	382 (72.2%)	
≥5 ng/mL	39 (32.3%)	147 (27.8%)	
Tumor location			0.097^a^
Colon	71 (55.9%)	284 (47.7%)	
Proximal	33 (26.0%)	129 (21.6%)	
Distal	38 (29.9%)	155 (26.0%)	
Rectum	56 (44.1%)	312 (52.3%)	
Synchronous or metachronous colorectal cancer	9 (7.1%)	52 (8.7%)	0.725
Extracolorectal cancer	5 (3.9%)	51 (8.6%)	0.098
Differentiation			0.184
Well	12 (10.3%)	82 (14.4%)	
Moderate	100 (85.5%)	422 (77.8%)	
Poorly	5 (4.3%)	44 (7.7%)	
Mucin production -> Mucinous adenocarcinoma	17 (13.4%)	40 (6.7%)	0.017
Lymphovascular or perineural invasion	66 (52.0%)	337 (56.5%)	0.376
MSI status			<0.001
MSI-H	24 (18.9%)	31 (5.2%)	
MSI-L	3 (2.4%)	23 (3.9%)	
MSS	100 (78.7%)	542 (90.9%)	
Tumor invasion^b^			0.229
T1	6 (4.7%)	37 (6.2%)	
T2	14 (11.0%)	67 (11.2%)	
T3	88 (69.3%)	439 (73.7%)	
T4	19 (15.0%)	53 (8.9%)	
Lymph node metastasis^b^			0.020
N0	63 (49.6%)	302 (50.7%)	
N1	23 (18.1%)	162 (27.2%)	
N2	41 (32.3%)	132 (22.1%)	
Pathologic stage^b^			0.593
I	17 (13.4%)	86 (14.4%)	
II	45 (35.4%)	204 (34.2%)	
III	47 (37.0%)	244 (40.9%)	
IV	18 (14.2%)	62 (10.4%)	

Group Y, patients ≤50 years; Group O, patients >50 years; CEA, carcinoembryonic antigen; MSI, microsatellite instability; MSI-H, high-frequency MSI; MSI-L, low-frequency MSI; MSS, microsatellite stability.

^a^Colon vs. rectum.

^b^Tumor invasion and pathologic stage were classified according to the criteria of the American Joint Committee on Cancer 7^th^ edition.

### Recurrence and survival

**Fig 1:**
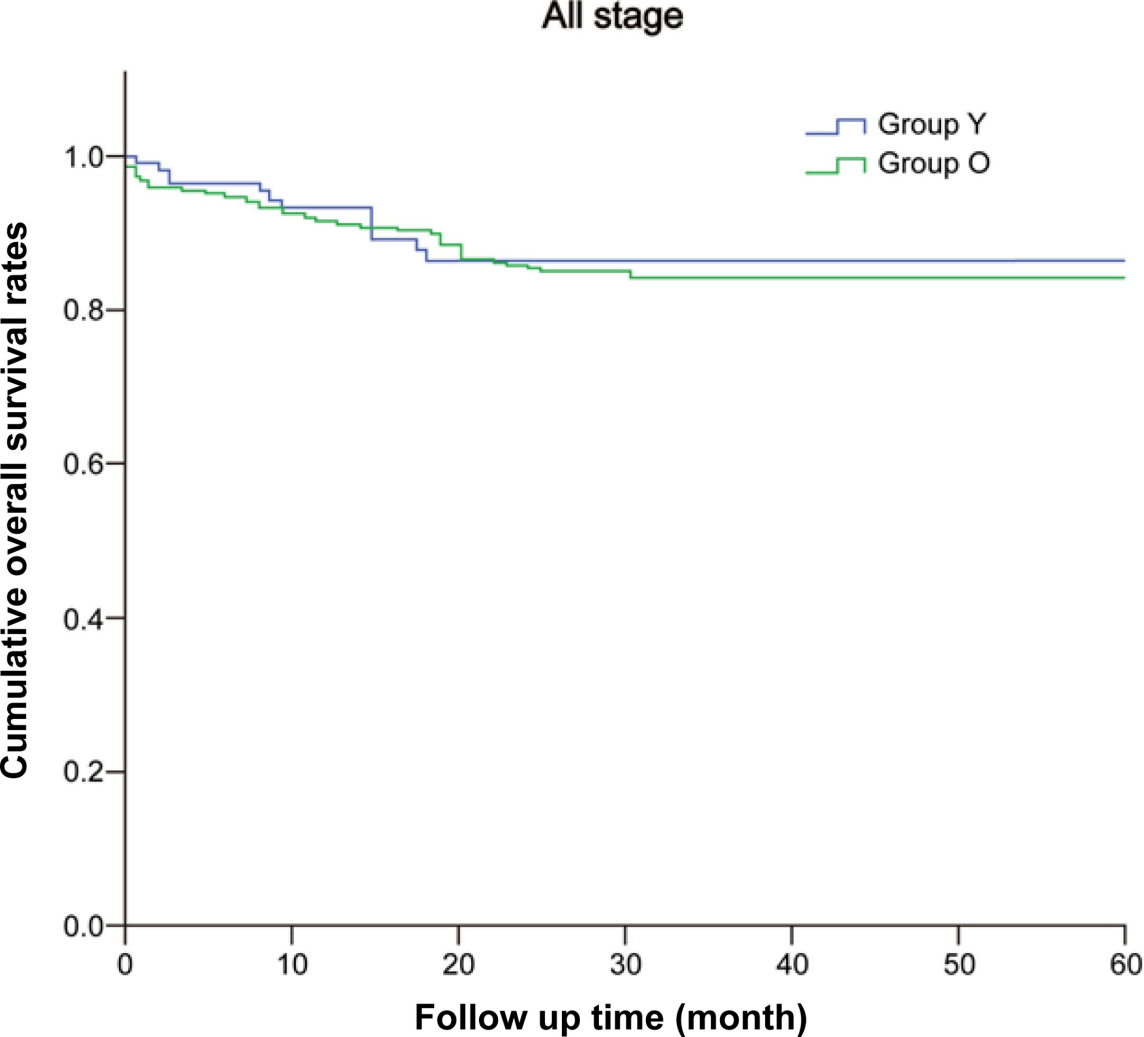
Five-year overall survival rates in Groups Y and O. There was no difference in 5-year overall survival significantly between Group Y and O. Group Y, patients ≤50 years; Group O, patients > 50 years (Group Y, 86.8%; Group O, 82.7%; *P* = 0.459).

**Fig 2:**
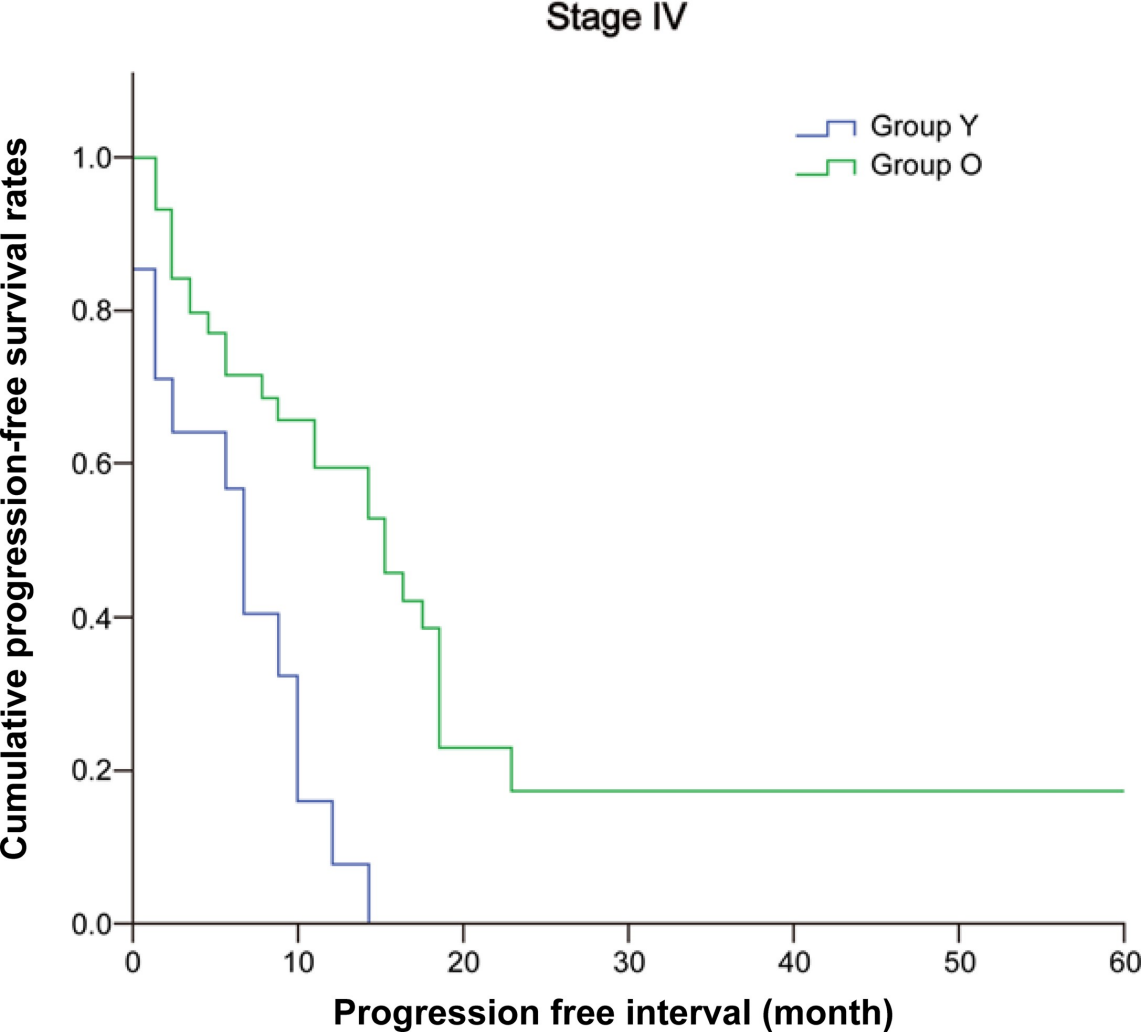
Progression-free survival in patients with stage IV colorectal cancer. The median progression-free survival in stage IV cases was significantly shorter in Group Y. Group Y, patients ≤50 years; Group O, patients >50 years (Group Y (8 months) vs. Group O (14 months); *P* = 0.002).

During the follow-up, 15 patients (14.9%) in Group Y and 94 (173%) in Group O developed recurrence and/or metastases. The recurrence rates were similar in the two groups (*P* = 0.665). The 5-year overall survivals were similar in both groups (Group Y, 86.8% vs. Group O, 82.7%; *P =* 0.459) (Fig 1). The five-year disease-free survival rates were also similar (81.5% in Group Y vs. 75.9% in Group O, *P* = 0.511) (Table 2). Although stage-to-stage comparisons also revealed no statistical differences, the median progression-free survival in stage IV cases was significantly shorter in Group Y (8 months in Group Y vs. 14 months in Group O, *P* = 0.002; Fig 2 and Table 2).

**Table 2:** Comparison of overall and disease-free survival in Groups Y and O with colorectal cancer.

	All patients (n = 723)	Group Y (n = 127)	Group O (n = 596)	P value
5-year overall survival	85.0%	86.8%	82.7%	0.459
Stage I	97.8%	100%	97.4%	0.551
Stage II	92.5%	100%	90.8%	0.082
Stage III	82.2%	88.8%	81.0%	0.593
Stage IV	38.8%	26.4%	41.3%	0.914
5-year disease-free survival	76.9%	81.5%	75.9%	0.511
Stage I	97.0%	85.7%	98.6%	0.255
Stage II	86.0%	94.7%	83.6%	0.351
Stage III	62.1%	65.7%	61.3%	0.983
Median progression-free survival in stage IV patients	10 months	8 months	14 months	0.002

Group Y, patients ≤50 years; Group O, patients >50 years.

By univariate analysis, cellular differentiation, lymphovascular/perineural invasion, pathological stage, and preoperative carcinoembryonic antigen (CEA) level were predictive factors for overall survival. Age and MSI status were not a significant prognostic factor. Multivariate analysis indicated that cellular differentiation and pathological stage were significant prognostic factors for overall survival in all patients. These two factors were equally significant in both groups (Table 3).

**Table 3:** Multivariate analyses of the prognostic factors for 5-year overall survival.

	Multivariate	
	HR (95% CI)	P value
Preoperative CEA ≥5 ng/mL	1.715 (0.980–3.000)	0.059
Differentiation		
Well	1.000	
Moderate	2.373 (0.573–9.830)	0.233
Poorly	8.604 (1.927–38.420)	0.005
Lymphovascular or perineural invasion	1.541 (0.833–2.851)	0.168
Pathological stage		
I	1.000	
II	2.509 (0.316–19.900)	0.384
III	3.829 (0.498–29.442)	0.197
IV	20.272 (2.566–160.147)	0.004

Group Y, patients ≤50 years; Group O, patients >50 years; HR, hazard ratio; CI, confidence interval; CEA, carcinoembryonic antigen; MSI, microsatellite instability; MSI-H, high-frequency MSI; MSI-L, MSI low-frequency; MSS, microsatellite stability; LN, lymph node.

## Discussion

In the present study, we noted a higher incidence of mucin production as well as a significantly higher MSI-H ratio in Group Y. The proportion of cases with N2 stage was also significantly higher in Group Y. There was no difference in the 5-year overall survival or disease-free survival between groups. However, the median progression-free survival in stage IV cases was shorter (8 months in Group Y and 14 months in Group O). This indicates that, although we did not note a difference in survival between groups, younger patients have more aggressive CRC than elderly patients in the advanced stages.

The characteristics of sporadic CRC in young patients remain unknown because these patients are older than pediatric patients and younger than elderly patients [16]. Studies on young CRC patients have used different criteria due to the ambiguous definition of “young” age. Some studies considered younger patients as those aged less than the screening age for CRC based on treatment guidelines [7,17], while others evaluated patients 10 years younger than screening age for CRC [15,18,19]. Thus, there is a need for a consensus about what age can be defined as young in CRC patients.

Rho et al. compared CRC of young (18–44 years) and late (age >44 years) onset [15] and reported that young-onset CRC patients had more aggressive disease, although the treatment patterns and survival outcomes were similar to those of the late-onset group. Segev et al. classified patients using a cut-off age of 50 years, reporting that left-sided CRC was dominant in both groups [20]. Thus, they suggested that patients should be offered sigmoidoscopy at 40 years of age. If there is a negative finding, another follow-up is needed at 45 years of age, and screening colonoscopy is recommended at 50 years of age. The mean age at diagnosis of CRC was 65 years; therefore, the recommended screening age is 50 years for the general population. Thus, we defined young patients as those aged <50 years, according to the guidelines for CRC screening [21]. However, diagnosis age <50 years was not a significant prognostic factor for survival in multivariate analysis, even though we hypothesized that the age at diagnosis could be a significant factor.

Mucinous adenocarcinoma (MUC) accounts for about 10% of all CRC cases [22]. MUCs are common among younger patients [22,23] and some studies have suggested that MUCs have a worse prognosis than non-MUCs [24,25]. However, other studies have reported that mucinous histology is not an independent prognostic factor for survival [26,27]. They suggested that the worse overall survival was due to the presence of more advanced disease stage in MUCs rather than the mucinous histology itself. In this study, Group Y patients had a higher proportion of mucin production than Group O, although there were no differences in pathological stage and survival. Thus, there is a lack of consensus about MUCs and we cannot draw conclusions about the relationship between prognosis and mucin production.

MSI is a hallmark of hereditary nonpolyposis colorectal carcinoma. MHI-H cancer can develop as sporadic or hereditary cancer and is found in approximately 10–15% of sporadic CRC cases [28,29]. Recent studies reported the proportion of MSI to be 19.7–41.0% in young CRC patients, depending on the age of onset [8]. Most studies revealed a better prognosis in patients with MSI-H CRC than in patients with MSS CRC. MSI-H CRC has several characteristics such as its localization in the proximal colon and its propensity to grow into large tumors. In young patients, MSI-H is associated with Lynch syndrome. These patients show mucinous and signet-ring cell differentiation [30]; however, patients with MSI-H have a favorable prognosis with a low frequency of distant metastasis and they have about 10% higher 5-year overall survival than patients with MSS [16,28,29,31,32]. Although most patients showed a high proportion of MSS, the ratio of MSI-H in this study was significantly higher in Group Y than in Group O (18.9% vs. 5.2%). Similar to that of mucin production, the high proportion of MSI-H in Group Y did not affect survival or prognosis.

The National Cancer Database reported that young-onset CRC more frequently exhibited a mucinous and signet-ring cell histology than later-onset CRC (12.6% vs. 18%; P < 0.001) [7]. However, the causes of these histological differences remain unknown. Moreover, young patients were likely diagnosed with advanced stage CRC because their symptoms were not recognized and evaluated. Surveillance, Epidermiology, and end Results Program (SEER) data from 1991 to 1999 revealed that young patients (20–40 years) with CRC had a poorer overall survival than elderly patients (60–80 years) (61.5% vs. 64.9%; p = 0.02) [7]. However, another study reported that stage-specific survival rates in young patients were equal to or exceeded those in elderly patients [33]. In our study, stage-specific survival was similar in both groups; There was no significant differences in overall survival or disease-free survival according to stage I, II or III. However, median progression-free survival differed significantly in stage IV cases. It demonstrated patients with stage IV in CRC showed worse prognosis than elderly with statistical significance. In the multivariate analysis, stage IV was significant prognostic factor for survival. We could not assess differences between age groups in stage IV separately because the sample size was very small (about 10% in each group). Further large-scale and multicenter studies are needed to understand the underlying causes of the differences in outcomes such as progression-free survival.

Until now, there has been a lack of adequate understanding of not only the criteria to define young adult CRC patients but also of their survival outcomes. CRC has heterogeneous features and researchers have generally focused on elderly patients with sporadic CRC and young patients with hereditary CRC. However, the proportion of young patients with sporadic CRC has increased. Most young patients are diagnosed with symptoms such as hematochezia or obstruction when the disease has already progressed. Elderly patients usually tend to be diagnosed when they undergo recommended screening after reaching a specific age. Thus, we need to identify common features among young patients through studies focused on these patients with sporadic CRC. We should also consider this population separately in terms of treatment and prognosis.

This study has several limitations. First, although this study compared a large number of young and elderly patients, it was performed in a single institution. This cohort may not represent the characteristics of young patients in CRC among the whole population. Second, there were differences in the distribution of the numbers of patients in this study because only 17% were young. We defined age <50 years as young in CRC patients according to treatment guidelines. However, age <40 or <45 years could also show definite discrepancies compared to age >50 years. Thus, it is necessary to compare patient groups depending on the age at diagnosis in 10-year increments rather than based on a specific age in a large-scale cohort.

Because this study was retrospective, we excluded patients with hereditary CRC based on the medical records. However, we might have overlooked family history in some patients. Thus, multicenter prospective studies of diverse cohorts are needed to verify features according to the age at diagnosis, familial components, and biomolecular characteristics.

## Conclusion

Although there were no differences in overall survival and disease-free survival between the groups, differentiation and pathological stage were significant prognostic factors for overall survival in multivariate analysis. The proportion of cases at each stage also did not differ; however, young patients with advanced stages showed a more aggressive prognosis. Further studies are necessary to understand the disease characteristics in young CRC patients, including the factors leading to differences in clinical and biological features between age groups. The findings of these studies will help improve treatment strategies and, ultimately, outcomes in CRC patients. We hope that our study findings encourage further clinical and biological studies to confirm the differences in biological behaviors of CRC between young and elderly patients.
